# Supplementary material for: Cross-membrane cooperation among bacteria can facilitate intracellular pathogenesis
Source: Nat Commun. 2025 Aug 11;16:7419. doi: 10.1038/s41467-025-62575-3 (PMC12339937; doi:10.1038/s41467-025-62575-3)
Supplement: Supplementary file 1 — Supplementary Information [file 41467_2025_62575_MOESM1_ESM.pdf]

Supp. Figure 1

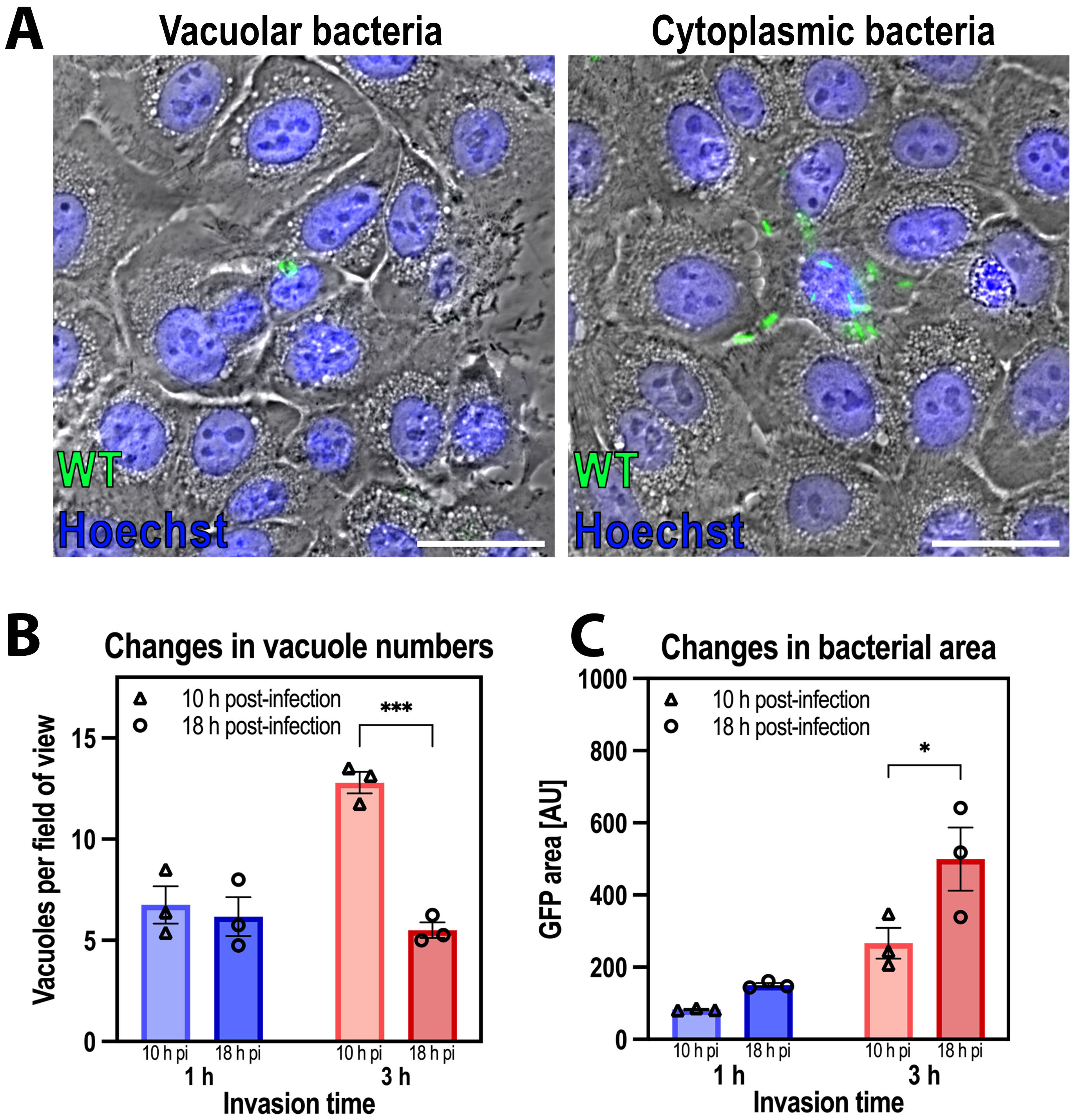

Supp. Figure 1: Comparison of the 1 and 3 h invasion time model

(A) Representative wide field phase contrast microscopy images of human corneal epithelial cells (hTCEpi) infected with *P. aeruginosa* PAO1 containing a plasmid for inducible GFP expression (pBAD-GFP) using MOI 10. Images were taken 5 h post-infection using a 100x oil-immersion objective. Bacteria (green), Hoechst (blue). Scale bars equal to 20 μm. (B/C) Image analysis of timelapse images measuring vacuole numbers (B) and bacterial area (C) of *P. aeruginosa* PAO1 wt at 10 and 18 h post-infection comparing the 1 and 3 h invasion time models. Data in graph B are re-plotted from Figure 1B. Data of biological replicates represented as mean ± SEM, N=3 (B/C). For statistical analysis, a Two-way ANOVA with multiple comparisons was performed. Exact P-values – (B) = 0.0002, (C) = 0.0192. $P \leq 0.05$ = *, $P \leq 0.001$ = ***. Source data are provided as a Source Data file.

Supp. Figure 2

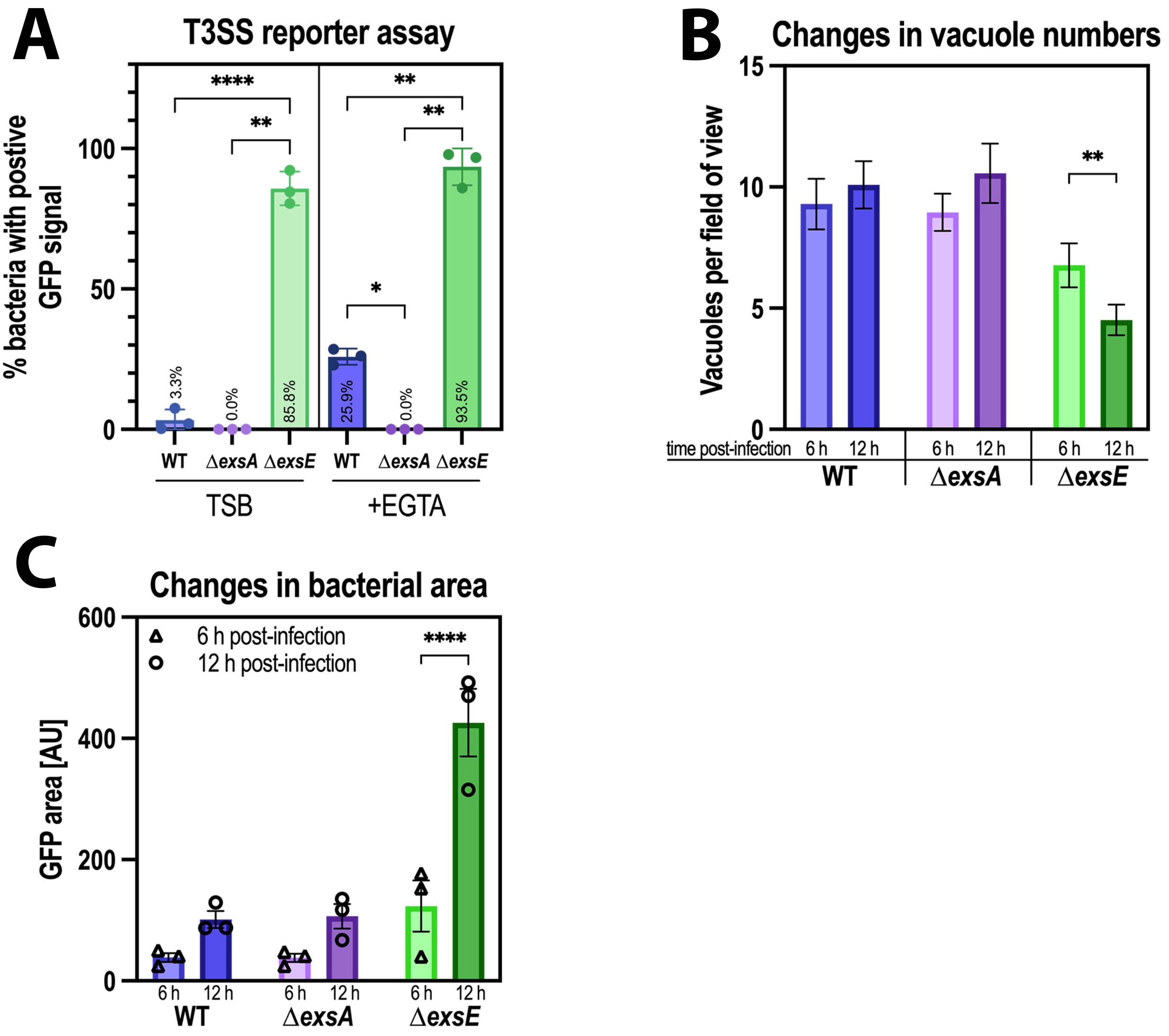

Supp. Figure 2: Constitutive expression of T3SS decreases vacuole numbers and increases bacterial area.
(A) Quantification of T3SS-expressing (GFP positive) bacteria in a spotting assay comparing *P. aeruginosa* PAO1 wild type (WT), Δ*exsA*, and Δ*exsE* under non-inducing (TSB) and inducing (+EGTA) conditions. (B/C) Image analysis of timelapse images measuring vacuole numbers (B) and bacterial area (C) at 6 and 12 h post-infection comparing P. *aeruginosa* PAO1 wild type (WT), Δ*exsA*, and Δ*exsE*. Data in graph B are re-plotted from Figure 3C. Data of biological replicates represented as mean ± SD (A) or mean ± SEM (B/C), N=3 (A/C), N=8 (B). For statistical analysis, a Two-way ANOVA with multiple comparisons was performed. Exact P values – (A): TSB WT vs. Δ*exsE* = >0.0001, TSB Δ*exsA* vs. Δ*exsE* = 0.0063, EGTA WT vs. Δ*exsA* = 0.0144, EGTA WT vs. Δ*exsE* = 0.0049, EGTA Δ*exsA* vs. Δ*exsE* = 0.0064; (B) = 0.0471; (C) = >0.0001. P ≤ 0.05 = *, P ≤ 0.01 = **, P ≤ 0.0001 = ****. Source data are provided as a Source Data file.

Supp. Figure 3

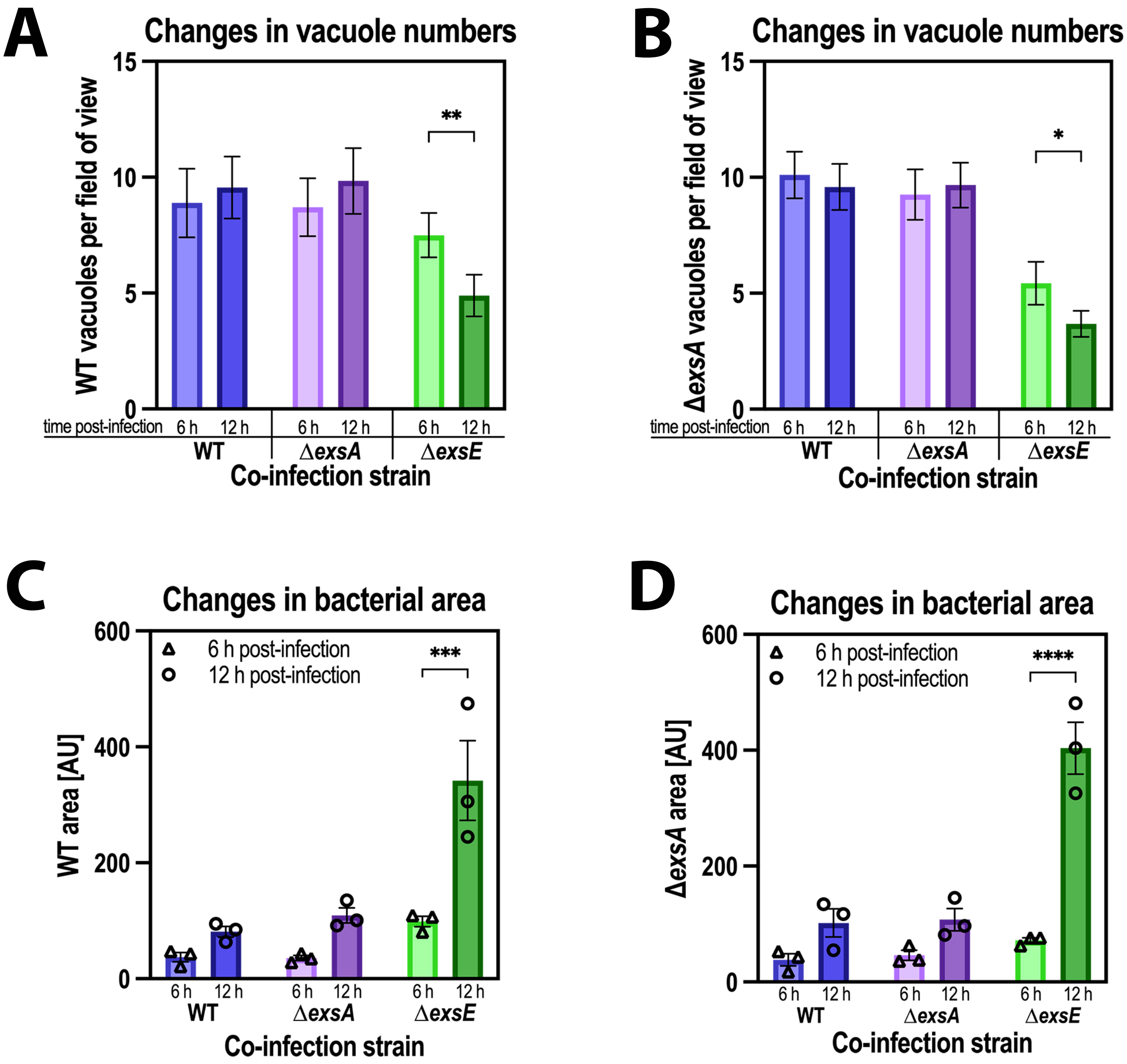

Supp. Figure 3: Co-infections with a T3SS^{ON} strain impact *P. aeruginosa* wild type/Δ*exsA* vacuoles and bacterial area. (A/B) Image analysis of timelapse images measuring vacuole numbers of *P. aeruginosa* PAO1 wild type (WT) (A) or Δ*exsA* (B) at 6 and 12 h post-infection, comparing co-infection of PAO1 WT/Δ*exsA* with WT, Δ*exsA*, or Δ*exsE*. Data in graphs A and B are re-plotted from Figures 4A and 4B, respectively. (C/D) Image analysis of timelapse images measuring bacterial area of *P. aeruginosa* PAO1 wild type (WT) (C) or Δ*exsA* (D) at 6 and 12 h post-infection, comparing co-infection of PAO1 WT/Δ*exsA* with WT, Δ*exsA*, or Δ*exsE*. Data represented as mean ± SEM, N=8 (A/B), N=3 (C/D). For statistical analysis, a Two-way ANOVA with multiple comparisons was performed. Exact P values – (A) = 0.0015; (B) = 0.0172; (C) = 0.0002; (D) = >0.0001. P ≤ 0.05 = *, P ≤ 0.01 = **, P ≤ 0.001 = ***, P ≤ 0.0001 = ****. Source data are provided as a Source Data file.

Supp. Figure 4

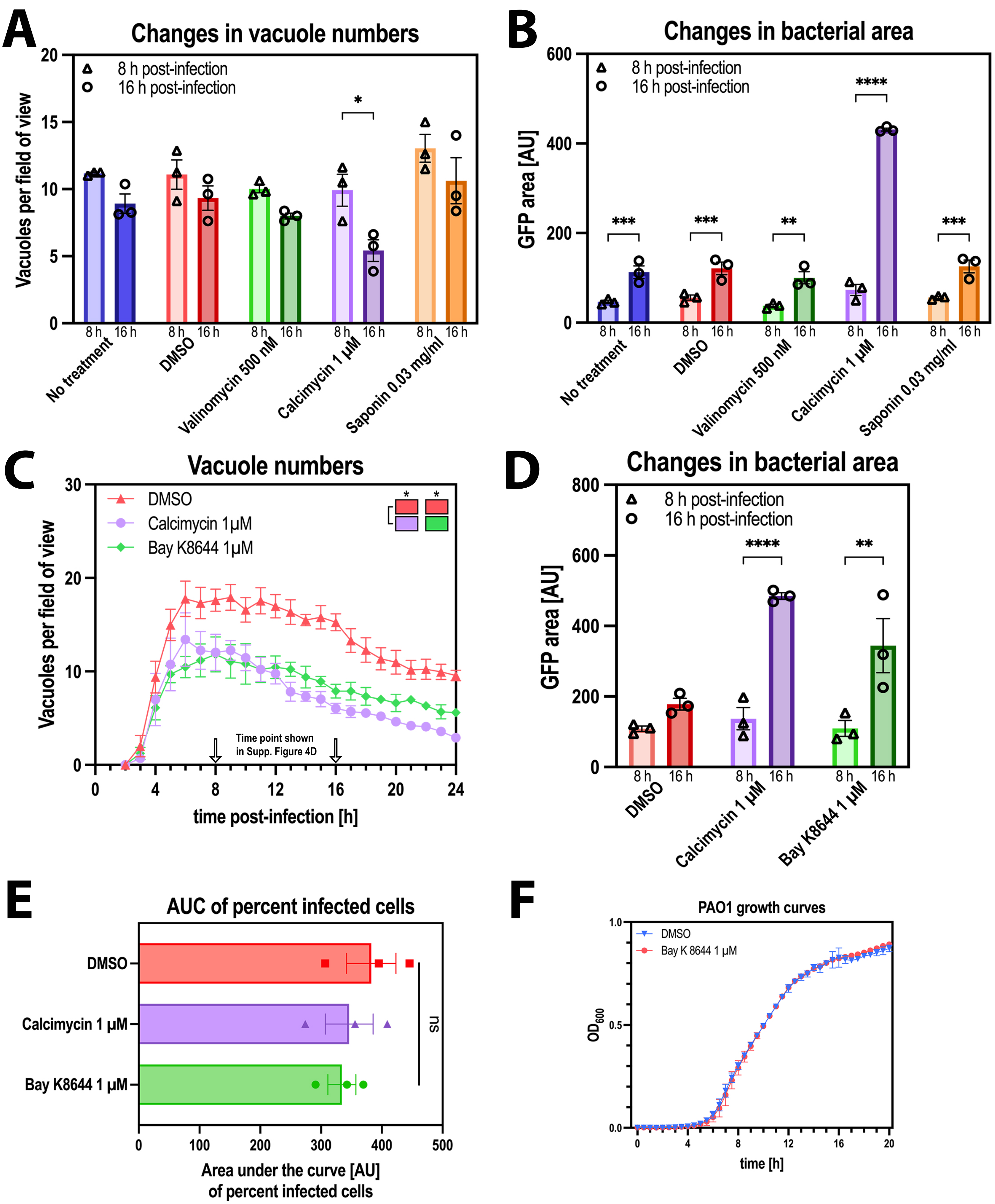

Supp. Figure 4: Compounds promoting Ca^{2+} influx influence vacuole numbers and bacterial area.

(A/B) Image analysis of timelapse images measuring vacuole numbers (A) and bacterial area (B) of *P. aeruginosa* PAO1 wild type at 8 and 16 h post-infection when treated with different compounds. Data in graph A are re-plotted from Figure 6A. (C/D/E) Image analysis of timelapse images measuring vacuole numbers (A), bacterial area (B), and percent infected cells represented as Area Under the Curve (AUC) bar plots (E) of *P. aeruginosa* PAO1 wild when treated with different compounds known to induce Ca^{2+} influx. (F) Growth of PAO1 wild type measured at OD_{600} in the presence of different compounds known to induce Ca^{2+} influx. Data represented as mean ± SEM (A-E) or mean ± SD (F), N=3. For statistical analysis, a Two-way ANOVA with multiple comparisons (A-D) or a One-way ANOVA with multiple comparisons (E) was performed. Exact P values – (A) = 0.0143; (B): 8h vs. 16h, No treatment = 0.0007, DMSO = 0.0008, Valinomycin = 0.0014, Calcimycin = >0.0001, Saponin = 0.0004; (C): DMSO vs. Calcimycin = 0.0118, DMSO vs. Bay K8644 = 0.0189; (D): 8h vs. 16h, Calcimycin = 0.0001, Bay K8644 = 0.0018. P ≤ 0.05 = *, P ≤ 0.01 = **, P ≤ 0.001 = ***, P ≤ 0.0001 = ****. Source data are provided as a Source Data file.
