# Supplementary material for: Cross-membrane cooperation among bacteria can facilitate intracellular pathogenesis
Source: Nat Commun. 2025 Aug 11;16:7419. doi: 10.1038/s41467-025-62575-3 (PMC12339937; doi:10.1038/s41467-025-62575-3)
Supplement: Supplementary file 2 — Description of Additional Supplementary Files [file 41467_2025_62575_MOESM2_ESM.pdf]

Description of Additional Supplementary Files:

Supplementary Movie 1: Representative wide field microscopy time lapse videos comparing 1 and 3 h invasion time Representative wide field microscopy time lapse video of humane corneal epithelial cells (hTCEpi) infected with P. aeruginosa PAO1 with a plasmid for inducible GFP expression using MOI 10. Extracellular bacteria were killed using Amikacin (200 µg/ml) before inducing GFPexpression in intracellular bacteria. Videos start at 4 h post-infection and show the difference in bacterial behavior between 1 and 3 h invasion time. Bacteria (green), Hoechst (gray). Images were taken using a 40x air objective and the scale bars represent 50 µm.

Supplementary Movie 2: Representative *in vivo* confocal microscopy videos of infected murine corneal epithelium Representative *in vivo* confocal microscopy videos of murine corneal epithelium (ROSAmT/mG mice, dtTomato cell membranes, red) from apical to basal side of the corneal epithelium infected with different combinations of fluorescent (green) and non-fluorescent bacteria (Δ*exsA*-GFP+10% Δ*exsA*, 90% Δ*exsA*GFP+10% Δ*exsE*, 10% Δ*exsE*-GFP). Images were taken 20 hours post-infection using the murine scratchinfection model at infection foci within the corneal epithelium using a 60x water-immersion objective. The videos first show a 3-D view of the infected corneal epithelium (apical and basal side are indicated in the video), followed by an orthogonal projection of the stack moving from the apical to the basal side and back.
