# Supplementary material for: Cross-membrane cooperation among bacteria can facilitate intracellular pathogenesis
Source: Nat Commun. 2025 Aug 11;16:7419. doi: 10.1038/s41467-025-62575-3 (PMC12339937; doi:10.1038/s41467-025-62575-3)
Supplement: Supplementary file 5 — Reporting Summary [file 41467_2025_62575_MOESM5_ESM.pdf]

nature portfolio

Corresponding author(s): Suzanne Fleiszig

Last updated by author(s): Jul 17, 2025

Reporting Summary

Nature Portfolio wishes to improve the reproducibility of the work that we publish. This form provides structure for consistency and transparency in reporting. For further information on Nature Portfolio policies, see our Editorial Policies and the Editorial Policy Checklist.

Statistics

For all statistical analyses, confirm that the following items are present in the figure legend, table legend, main text, or Methods section.

n/a	Confirmed	
☐	☒	The exact sample size (n) for each experimental group/condition, given as a discrete number and unit of measurement
☐	☒	A statement on whether measurements were taken from distinct samples or whether the same sample was measured repeatedly
☐	☒	The statistical test(s) used AND whether they are one- or two-sided<br>*Only common tests should be described solely by name; describe more complex techniques in the Methods section.*
☒	☐	A description of all covariates tested
☐	☒	A description of any assumptions or corrections, such as tests of normality and adjustment for multiple comparisons
☐	☒	A full description of the statistical parameters including central tendency (e.g. means) or other basic estimates (e.g. regression coefficient) AND variation (e.g. standard deviation) or associated estimates of uncertainty (e.g. confidence intervals)
☐	☒	For null hypothesis testing, the test statistic (e.g. F, t, r) with confidence intervals, effect sizes, degrees of freedom and P value noted<br>*Give P values as exact values whenever suitable.*
☒	☐	For Bayesian analysis, information on the choice of priors and Markov chain Monte Carlo settings
☒	☐	For hierarchical and complex designs, identification of the appropriate level for tests and full reporting of outcomes
☒	☐	Estimates of effect sizes (e.g. Cohen's d, Pearson's r), indicating how they were calculated

Our web collection on statistics for biologists contains articles on many of the points above.

Software and code

Policy information about availability of computer code

Data collection	NIS Elements (version 5.21.01)<br>Olympus FV10-ASW (version 04.02)<br>Gen5 microplate reader software (v2.06)
Data analysis	Imaris software (v 10.1)<br>ImageJ (version 2.14.0/1.54f)<br>Graphpad Prism Version 10.5.0

For manuscripts utilizing custom algorithms or software that are central to the research but not yet described in published literature, software must be made available to editors and reviewers. We strongly encourage code deposition in a community repository (e.g. GitHub). See the Nature Portfolio guidelines for submitting code & software for further information.

Data

Policy information about availability of data

All manuscripts must include a data availability statement. This statement should provide the following information, where applicable:

- Accession codes, unique identifiers, or web links for publicly available datasets
- A description of any restrictions on data availability
- For clinical datasets or third party data, please ensure that the statement adheres to our policy

Plasmids and bacterial gene knockout mutants generated for this study are available upon request. All tools used for image analysis have been made publicly available on GitHub and are accessible through Zenodo (DOI: 10.5281/zenodo.15579800).

Research involving human participants, their data, or biological material

Policy information about studies with human participants or human data. See also policy information about sex, gender (identity/presentation), and sexual orientation and race, ethnicity and racism.

Reporting on sex and gender	This study does not involve human participants, their data or biological material.
Reporting on race, ethnicity, or other socially relevant groupings	This study does not involve human participants, their data or biological material.
Population characteristics	This study does not involve human participants, their data or biological material.
Recruitment	This study does not involve human participants, their data or biological material.
Ethics oversight	This study does not involve human participants, their data or biological material.

Note that full information on the approval of the study protocol must also be provided in the manuscript.

Field-specific reporting

Please select the one below that is the best fit for your research. If you are not sure, read the appropriate sections before making your selection.

☒ Life sciences ☐ Behavioural & social sciences ☐ Ecological, evolutionary & environmental sciences

For a reference copy of the document with all sections, see nature.com/documents/nr-reporting-summary-flat.pdf

Life sciences study design

All studies must disclose on these points even when the disclosure is negative.

Sample size	Prior publications from our lab on this topic suggested that all experiments should be performed minimum as biological triplicates.
Data exclusions	No data was excluded from analysis.
Replication	All the experiments have been replicated.
Randomization	Randomization was not necessary, because the data analysis was performed masked using a macro, thereby eliminating the impact of the researchers.
Blinding	Blinding was not necessary, because the data analysis was performed masked using a macro, thereby eliminating the impact of the researchers.

Reporting for specific materials, systems and methods

We require information from authors about some types of materials, experimental systems and methods used in many studies. Here, indicate whether each material, system or method listed is relevant to your study. If you are not sure if a list item applies to your research, read the appropriate section before selecting a response.

Materials & experimental systems

n/a	Involved in the study	
☒	☐	Antibodies
☐	☒	Eukaryotic cell lines
☒	☐	Palaeontology and archaeology
☐	☒	Animals and other organisms
☒	☐	Clinical data
☒	☐	Dual use research of concern
☒	☐	Plants

Methods

n/a	Involved in the study	
☒	☐	ChIP-seq
☒	☐	Flow cytometry
☒	☐	MRI-based neuroimaging

Eukaryotic cell lines

Policy information about cell lines and Sex and Gender in Research

Cell line source(s)	hTCEpi cells were obtained from Prof. Danielle Robertson, who first created this cell line. DOI to her original publication: https://doi.org/10.1167/iovs.04-0528
Authentication	Cells were originally obtained by Prof. Danielle Robertson who created the cell line (see above). Beyond that, cells were characterized base on cell morphology and growth behavior.
Mycoplasma contamination	The cells were routinely monitored for Mycoplasma contamination and were negative.
Commonly misidentified lines (See ICLAC register)	No commonly misidentified cell lines were used.

Animals and other research organisms

Policy information about studies involving animals; ARRIVE guidelines recommended for reporting animal research, and Sex and Gender in Research

Laboratory animals	8- to 16-week old mice ROSAmT/mG mice were used.
Wild animals	This study did not involve wild animals.
Reporting on sex	Male and female mice were used.
Field-collected samples	This study did not involve samples collected in the field.
Ethics oversight	All procedures involving animals were carried out in accordance with the standards established by the Association for the Research in Vision and Ophthalmology, under protocol AUP-2019-06-12322-1, approved by the Animal Care and Use Committee, University of California Berkeley.

Note that full information on the approval of the study protocol must also be provided in the manuscript.

Plants

Seed stocks	This study did not involve any plants.
Novel plant genotypes	This study did not involve any plants.
Authentication	This study did not involve any plants.
